# Supplementary material for: A co-crystal berberine-ibuprofen improves obesity by inhibiting the protein kinases TBK1 and IKKɛ
Source: Commun Biol. 2022 Aug 12;5:807. doi: 10.1038/s42003-022-03776-0 (PMC9374667; doi:10.1038/s42003-022-03776-0)
Supplement: Supplementary file 2 — Supplementary Information [file 42003_2022_3776_MOESM2_ESM.pdf]

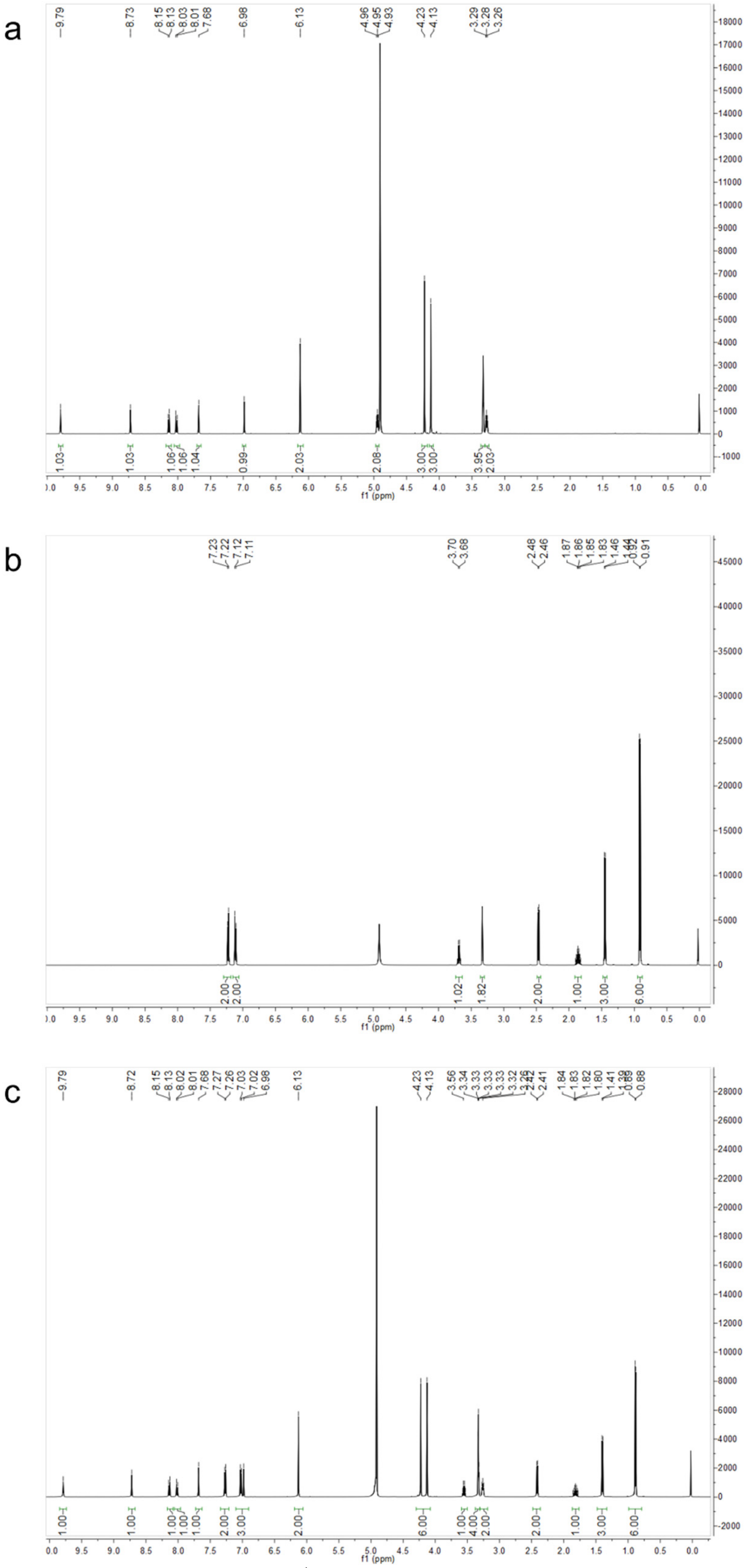

Supplementary Figure 1. ^{1}H NMR hydrogen spectra of BBR (BCl·$2H_2O$) (a), Ibu (b) and BJ (c)

a: ^{1}H NMR (500 MHz, MeOD) δ 9.79 (s, 1H), 8.73 (s, 1H), 8.14 (d, J = 9.1 Hz, 1H), 8.02 (d, J = 9.1 Hz, 1H), 7.68 (s, 1H), 6.98 (s, 1H), 6.13 (s, 2H), 4.97 – 4.92 (m, 2H), 4.23 (s, 3H), 4.13 (s, 3H), 3.33 (dt, J = 3.2, 1.6 Hz, 4H), 3.30 – 3.24 (m, 2H).

b: ^{1}H NMR (500 MHz, MeOD) δ 9.79 (s, 1H), 8.73 (s, 1H), 8.14 (d, J = 9.1 Hz, 1H), 8.02 (d, J = 9.1

Hz, 1H), 7.68 (s, 1H), 6.99 (s, 1H), 6.98 (s, 1H), 6.13 (s, 2H), 4.97 – 4.92 (m, 2H), 4.23 (s, 3H), 4.13 (s, 3H), 3.33 (dt, J = 3.2, 1.6 Hz, 4H), 3.30 – 3.24 (m, 2H).

c: ^{1}H NMR (500 MHz, MeOD) δ 9.79 (s, 1H), 8.73 (s, 1H), 8.14 (d, J = 9.1 Hz, 1H), 8.02 (d, J = 9.1 Hz, 1H), 7.68 (s, 1H), 6.99 (s, 1H), 6.98 (s, 1H), 6.13 (s, 2H), 4.97 – 4.92 (m, 2H), 4.23 (s, 3H), 4.13 (s, 3H), 3.33 (dt, J = 3.2, 1.6 Hz, 4H), 3.30 – 3.24 (m, 2H).

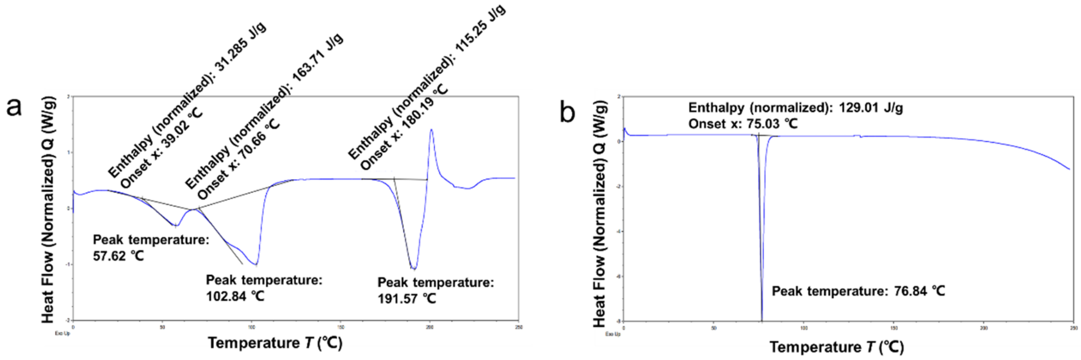

Supplementary Figure 2. DSC curves of BBR ($BCl\cdot 2H_2O$) (a) and Ibu (b).

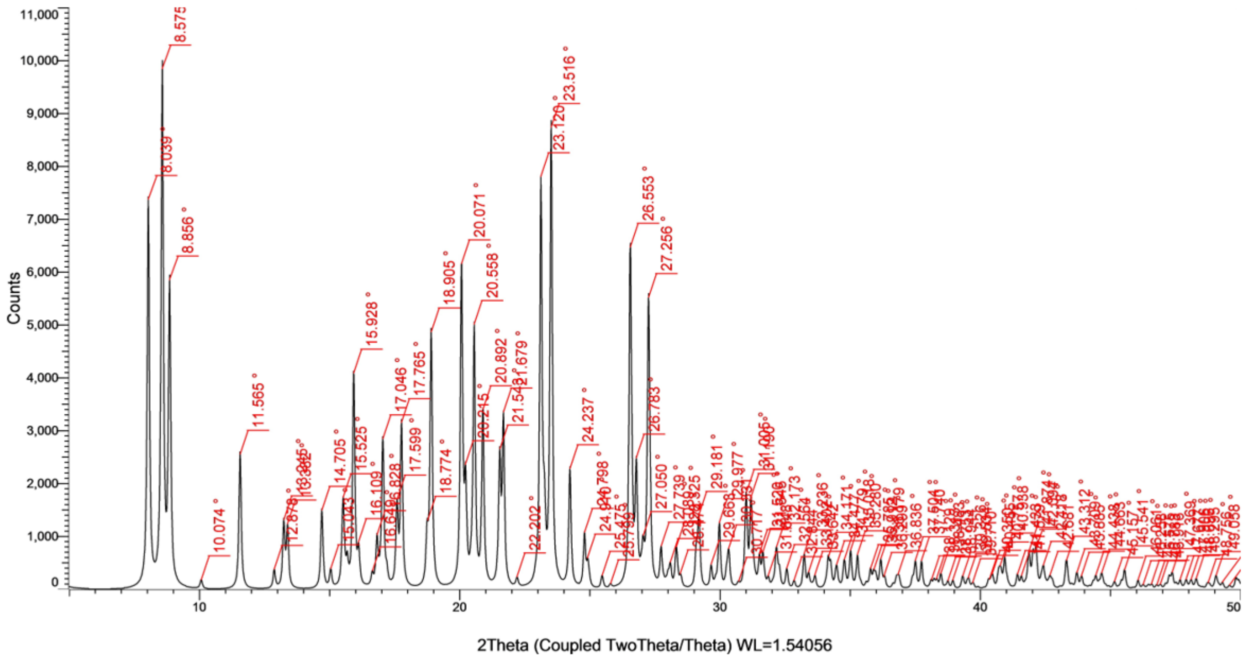

Supplementary Figure 3. The simulated PXRD patterns of corresponding BJ single crystal structure.

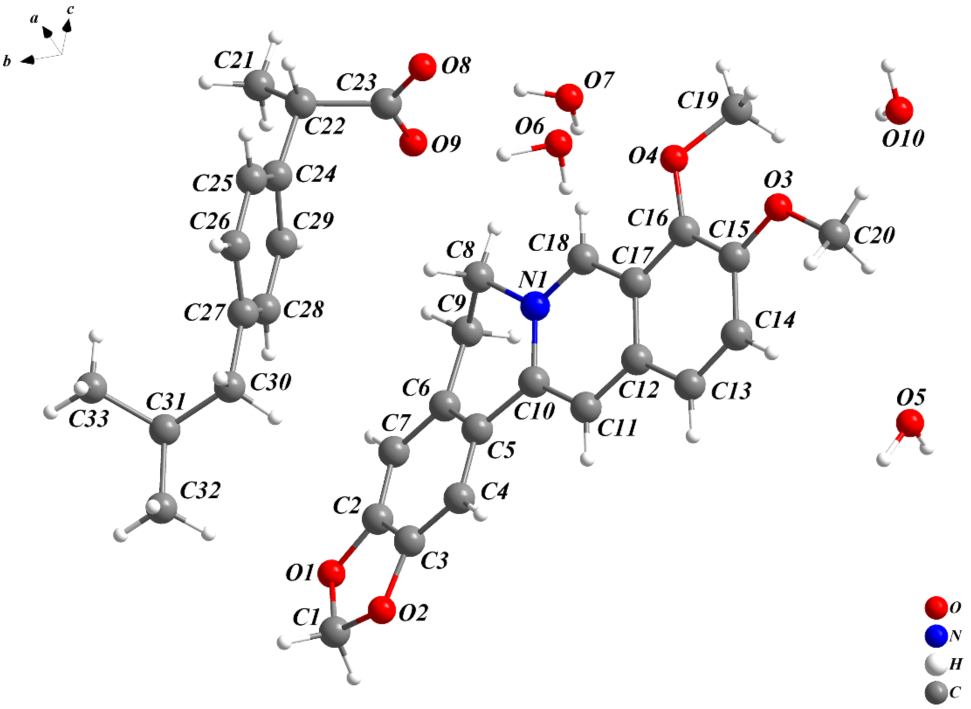

Supplementary Figure 4. Asymmetric structural unit of BJ.

Supplementary Table 1

Hydrogen bonding table for the co-crystal BJ

D---H....A [ARU]	d (D-H)	d (H....A)	d (D....A)	<D-H....A
O5--H5A..O7 [664]	0.78	1.97	2.738(5)	169
O5--H5B..O6[455]	0.87	1.92	2.780(5)	173
O6--H6A..O9[555]	1.02	1.73	2.718(5)	161
O6--H6B..O5[664]	0.92	1.81	2.695(6)	162
O7--H7A..O8[555]	0.89	1.82	2.700(5)	171
O7--H7B..O6[555]	1.01	1.82	2.743(5)	150
O10--H10A..O9[765]	0.87	1.95	2.800(6)	166
O10--H10B..O8[764]	0.87	2.05	2.915(6)	172
C11--H11..O10[664]	0.95	2.47	3.398(6)	164
C13--H13..O9[455]	0.95	2.5	3.324(6)	145
C18--H18..O7[555]	0.95	2.39	3.307(6)	162
C19--H19C..O5[665]	0.98	2.5	3.456(6)	164
C33--H33B..O2[565]	0.98	2.59	3.572(18)	177

Translation of ARU-Code to CIF and Equivalent Position Code:

#1 [455] = -1+x,y,z

#2 [565] = 1/2+x,3/2-y,z

#3 [664] = 1-x,1-y,-1/2+z

#4 [665] = 1-x,1-y,1/2+z

#5 [764] = 2-x,1-y,-1/2+z

#6 [765] = 2-x,1-y,1/2+z

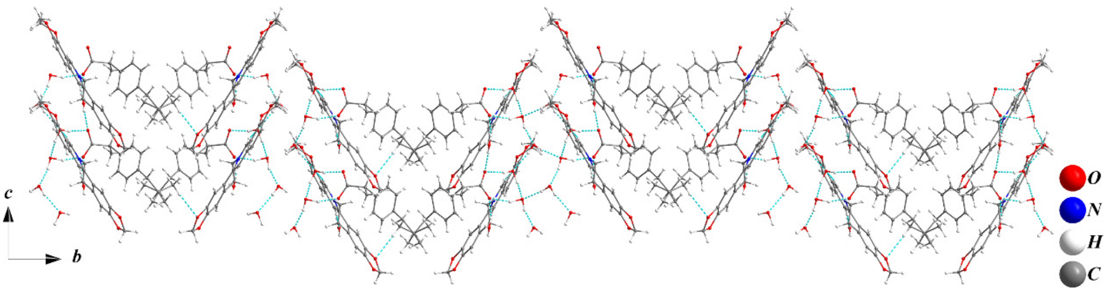

Supplementary Figure 5. Crystal packing of BJ viewed along the a-axis (hydrogen bonds are represented by light blue dotted line).

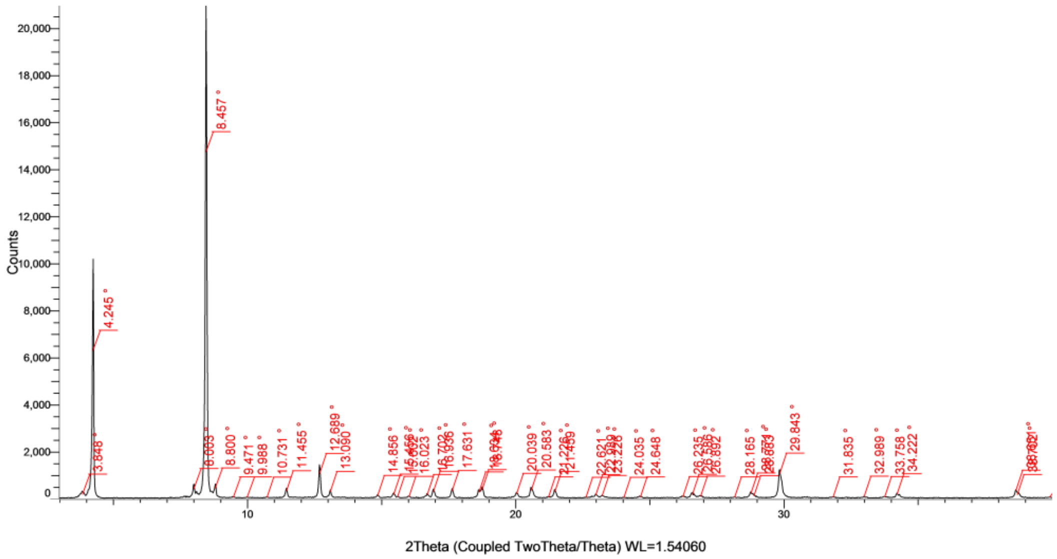

Supplementary Figure 6. The PXRD patterns of BJ after DVS.

Supplementary Table 2

NO.	Standard Buffer Solutions	pH	Composition
1	HCl solution	1.0	Pipette 4.55 mL of hydrochloric acid (37% HCl) into 500 mL deionized water. Mix well.
2	Acetate buffer	4.5	Place 1.495 g sodium acetate trihydrate in a 500 mL volumetric flask, add 7 mL 2 N acetic acid, then add deionized water to volume, and mix well.<br>If pH>4.5, please add suitable amount of 2 N acetic acid to adjust pH value to 4.5.<br>If pH<4.5, please add suitable amount of sodium acetate solution to adjust pH value to 4.5.<br>Record the actual pH.<br>* 2N acetic acid: Add 11.5 mL glacial acetic acid into a 100 mL volumetric flask, then add deionized water to volume, and mix well.
3	Phosphate buffer	6.8	Place 125 mL of the monobasic potassium phosphate solution in a 500 mL volumetric flask, add 56 mL of the 2 N sodium hydroxide solution, then add deionized water to volume.<br>* 2 N sodium hydroxide: Weigh 16 g NaOH in a 200 mL plastic volumetric flask, add deionized water to volume and mix well.<br>* Potassium Phosphate, Monobasic 0.2 M: Dissolve 5.444 g of monobasic potassium phosphate (KH_2PO_4) in water, and dilute with deionized water to 200 mL.
4	SGF	2.0	Step 1: Weigh 2 g of sodium chloride (NaCl) and 1 g of Triton X-100

			into a 1000 mL flask followed by addition of about 800 mL of deionized water. The mixture is stirred until all solids are dissolved. Add about 100 mL of 0.1 N HCl, check pH value with a pH meter and adjust the pH to 2.0 with HCl (1 N) or NaOH (1 N). Then dilute to the volume with deionized water. * 1N sodium hydroxide: Weigh 8 g NaOH in a 200 mL plastic volumetric flask, add deionized water to volume and mix well.
5	FaSSIF-v1	6.5	Step 1: Place 0.210 g sodium hydroxide, 2.235 g sodium dihydrogen phosphate and 3.093 g sodium chloride in a 500 mL volumetric flask, add about 0.450 L deionized water, and adjust the pH to 6.5 with 1N sodium hydroxide or 1 N hydrochloric acid. Make up to volume (0.5 L) with deionized water. Step 2: Place and dissolve 1.120 g FaSSIF/FeSSIF/FaSSGF instant powder with 0.250 L of buffer (from step 1) in a 500 mL volumetric flask, then add buffer (from step 1) to volume, and mix well.
6	FeSSIF-v1	5.0	Step 1: Place 2.020 g sodium hydroxide, 4.325 g acetic acid and 5.937 g sodium chloride in a 500 mL volumetric flask, add about 0.450 L deionized water, and adjust the pH to 5.0 with 1 N sodium hydroxide or 1 N hydrochloric acid. Make up to volume (500 mL) with deionized water. Step 2: Place and dissolve 5.600 g FaSSIF/FeSSIF/FaSSGF instant powder with 0.250 L of buffer (from step 1) in a 500 mL volumetric flask, then add buffer (from step 1) to volume, and mix well.
7	CMC-Na		Place 10.16949 g CMC-Na in a 1000 mL volumetric flask, add about 0.750 L deionized water. Make up to volume (1000 mL) with deionized water and placed overnight.

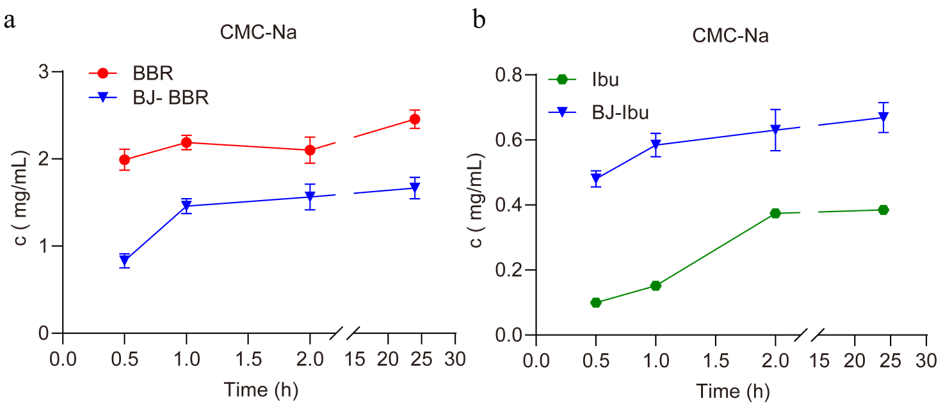

Supplementary Figure 7. Dynamic solubility of BBR ($BCl\cdot 2H_2O$) of BJ (a) and Ibu of BJ (b) in 0.5% CMC-Na.

Supplementary Table 3

The pH of the solution after 24 hours.

NO.	Standard Buffer Solutions	Blank vehicle （pH）	BBR ($BCl\cdot 2H_2O$)	Ibu	BJ
1	HCl solution	1.08±0.01	1.05±0.01	1.07±0.01	1.03±0.01
2	Acetate buffer	4.54±0.01	4.57±0.01	4.56±0.01	4.82±0.02
3	Phosphate buffer	6.84±0.01	6.90±0.02	6.60±0.02	6.90±0.01
4	SGF	2.03±0.01	2.07±0.01	2.00±0.01	5.38±0.01
5	FaSSIF-v1	6.51±0.01	6.54±0.01	6.04±0.01	5.50±0.02

6	FeSSIF-v1	5.04±0.01	5.03±0.01	5.05±0.01	5.17±0.01
7	CMC-Na	6.51±0.01	6.20±0.01	5.57±0.01	6.70±0.01

Supplementary Table 4

Pharmacokinetic parameters of BBR ($BCl\cdot 2H_2O$) and BJ in rats after oral administration (n = 5).

Pharmacokinetic parameters	BBR ($BCl\cdot 2H_2O$)	BJ
T_{max} (h)	0.500±0.08	3.800±1.304
C_{max} ($nmol\cdot mL^{-1}$)	1.638±0.132	2.907±0.121
$AUC_{(0-t)}$ ($nmol\cdot h\cdot mL^{-1}$)	5.005±0.974	24.639±0.493
$AUC_{(0-\infty)}$ ($nmol\cdot h\cdot mL^{-1}$)	9.965±7.746	30.750±3.421

Supplementary Table 5

Primers used for *qRT-PCR* Analysis.

Gene name	Forward primer sequence (5'–3')	Reverse primer sequence (5'–3')
Fasn	TGCCCGAGTCAGAGAACCTACAG	TCCATAGAGCCCAGCCTTCCATC
Acaca	CCCAGAGATGTTTCGGCAGTCAC	GTCAGGATGTCGGAAGGCAAAGG
Lpl	CGCTCTCAGATGCCCTACAAAGTG	TTGTGTTGCTTGCCATCCTCAGTC
Ucp1	GAAACACCTGCCTCTCTCGGAAAC	GCATTCTGACCTTCACGACCTCTG
Ppargc1a	GTGCCACCGCCAACCAAGAG	TTCCTCGTGTCCTCGGCTGAG
Ppargc1b	TGCTGGAGACTGCTCTGGAAGG	CCACTGCTGCTGCTGCTGTC
Cox8b	CCCCTATCCTGCGGCTGCTC	CGGCGGAAGTGGGAGTTTTGG
Elvol3	AGGATGCCACACAACAACGGAAC	CGGTGGAAGAAGTGAGCGAATAGG
Tnf-α	GCGACGTGGAACTGGCAGAAG	GCCACAAGCAGGAATGAGAAGAGG
Il-6	CTTCTTGGGACTGATGCTGGTGAC	AGGTCTGTTGGGAGTGGTATCCTC
Ccl2	CCACTCACCTGCTGCTACTCATTC	CTTCTTTGGGACACCTGCTGCTG
Adgre1	TTCCTGCTGTGTCGTGCTGTTC	GCCGTCTGGTTGTCAGTCTTGTC
Itgax	TGAGAGCCCAGACGAAGACAGTG	GATGATAGCCAGCAGCAGCAGAC
Tbk1	CAGTCTTCAGGACATCAGCAGCAG	CTGTCTCTTGGATGCGTGCCTTC
Ikbke	TGGTGTGACTGTGGATCTCTGGAG	GGCTGGCTTCTCTGTGGTGATTC
GAPDH	CAGTATGACTCCACTCACGGCAA	CTCGCTCCTGGAAGATGGTGAT

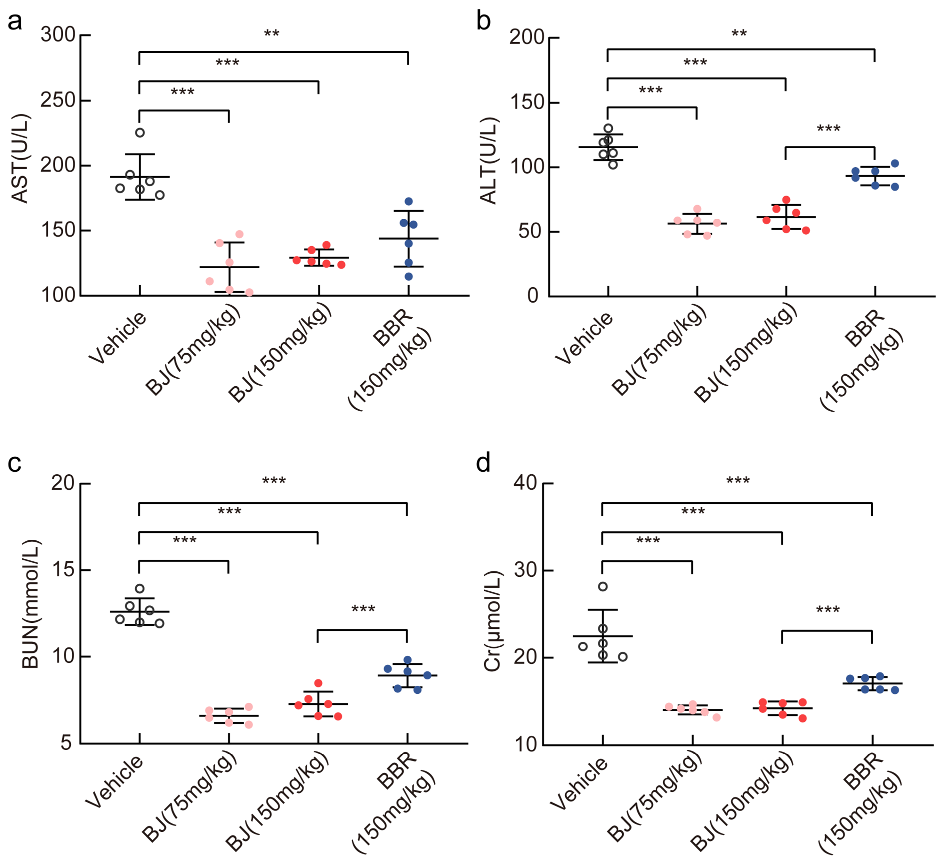

Supplementary Figure 8. (a-d) BJ or BBR does not induce db/db toxicity. Serum levels of ASAT, ALAT, CREA and BUN in db/db mice after treatment with BJ or BBR for 5 weeks (n=6 per group). Data are expressed as mean ± SD. NS = not significant, *P < 0.05, **P < 0.01, ***P < 0.001.

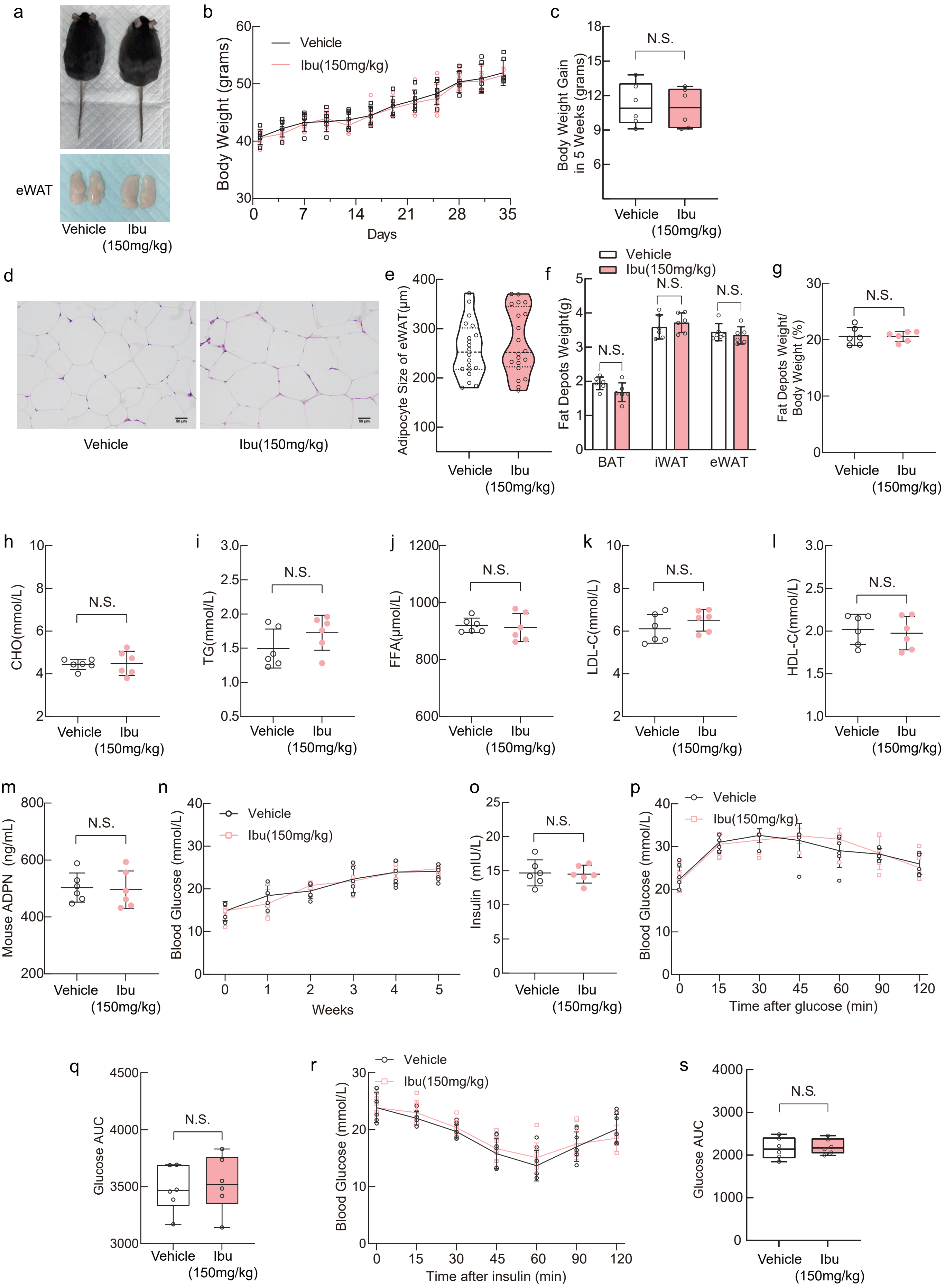

Supplementary Figure 9. (a) Representative images of db/db mice and their eWAT form db/db mice

treated with vehicle or Ibu. (b-c) Body weight of db/db mice in treatment groups (n=6 per group). (d-e) Representative HE staining images of epididymal adipose tissue (scale bar = 100 μm) and adipocyte size quantification. (f) Weight of BAT, iWAT and eWAT in different groups of db/db mice (n=6 per group) (g) Percentage of fat depots weight to the body weight (n=6 per group). (h-m) Serum levels of CHO, TG, FFA, LDL-C, HDL-C, and ADPN in the fasted state (n=6 per group). (n) Weekly fasting glucose levels of db/db mice (n=6 per group). (o) Fasting serum insulin levels in db/db mice after 35 days of treatment (n=6 per group). (p-q) GTT and the area under the curve (AUC) (n=6 per group). (r-s) ITT and the area under the curve (AUC) (n=6 per group). Data are expressed as mean ± SD. NS = not significant, *P < 0.05, **P < 0.01, ***P < 0.001.

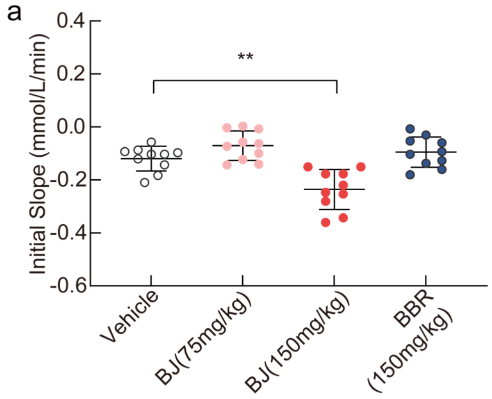

Supplementary Figure 10. (a) The initial slope of ITT (n=10 per group). Data are expressed as mean ± SD. NS = not significant, *P < 0.05, **P < 0.01, ***P < 0.001.

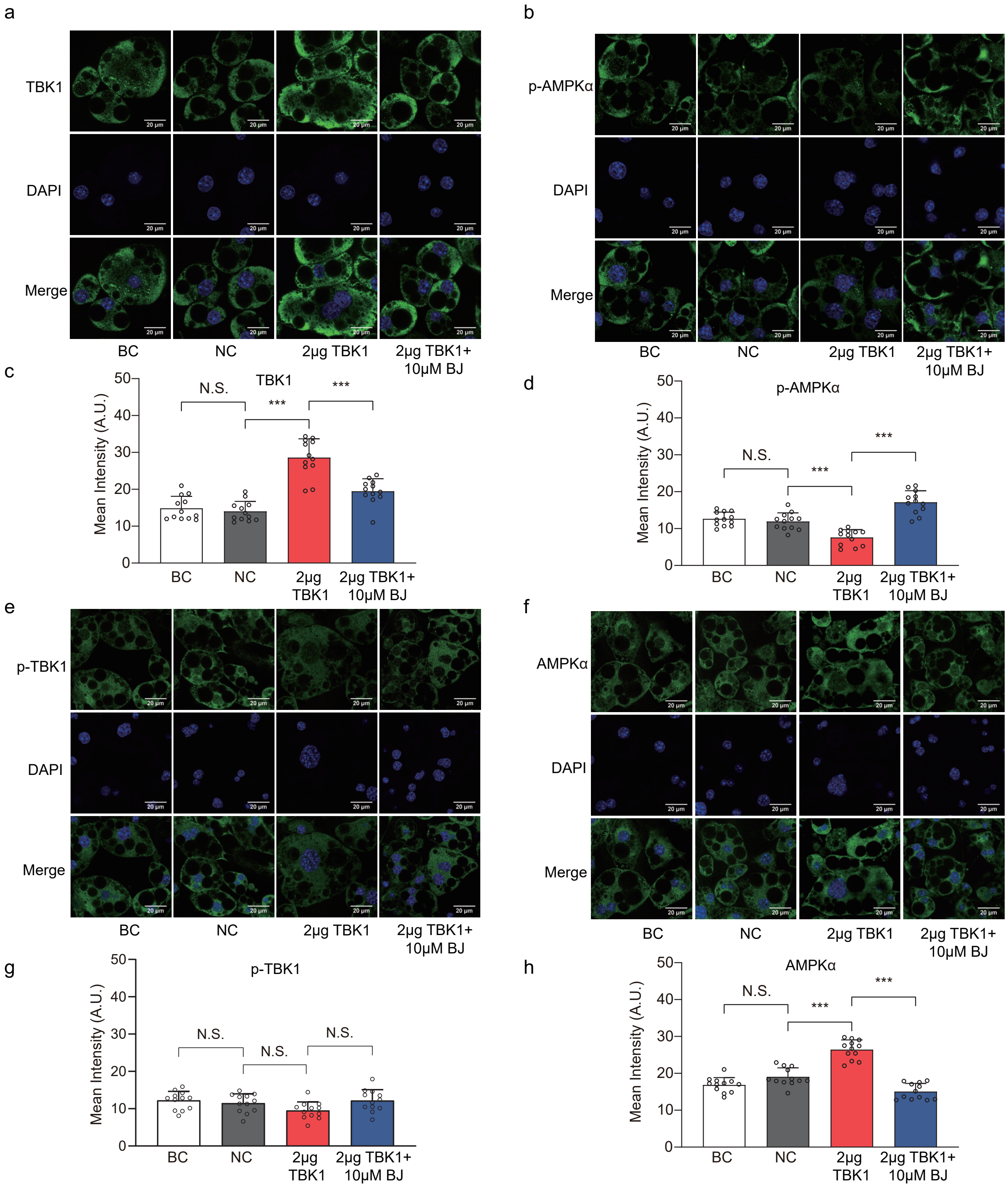

Supplementary Figure 11. (a-b) Immunofluorescence staining of TBK1 (a) and p-AMPKα (b) in differentiated 3T3-L1 adipocytes transfected with TBK1 plasmid or empty vector followed by treatment with or without BJ (scale bar = 20 μm). (c-d) Quantification of fluorescence intensity of TBK1 (c) and p-AMPKα (d) (n=12 per group). (e-f) Immunofluorescence staining of p-TBK1 (e) and

AMPKα (f) in differentiated 3T3-L1 adipocytes transfected with TBK1 plasmid or empty vector followed by treatment with or without BJ (scale bar = 20 μm). (g-h) Quantification of fluorescence intensity of p-TBK1 (g) and AMPKα (h) (n=12 per group). Data are expressed as mean ± SD. NS = not significant, *P < 0.05, **P < 0.01, ***P < 0.001.

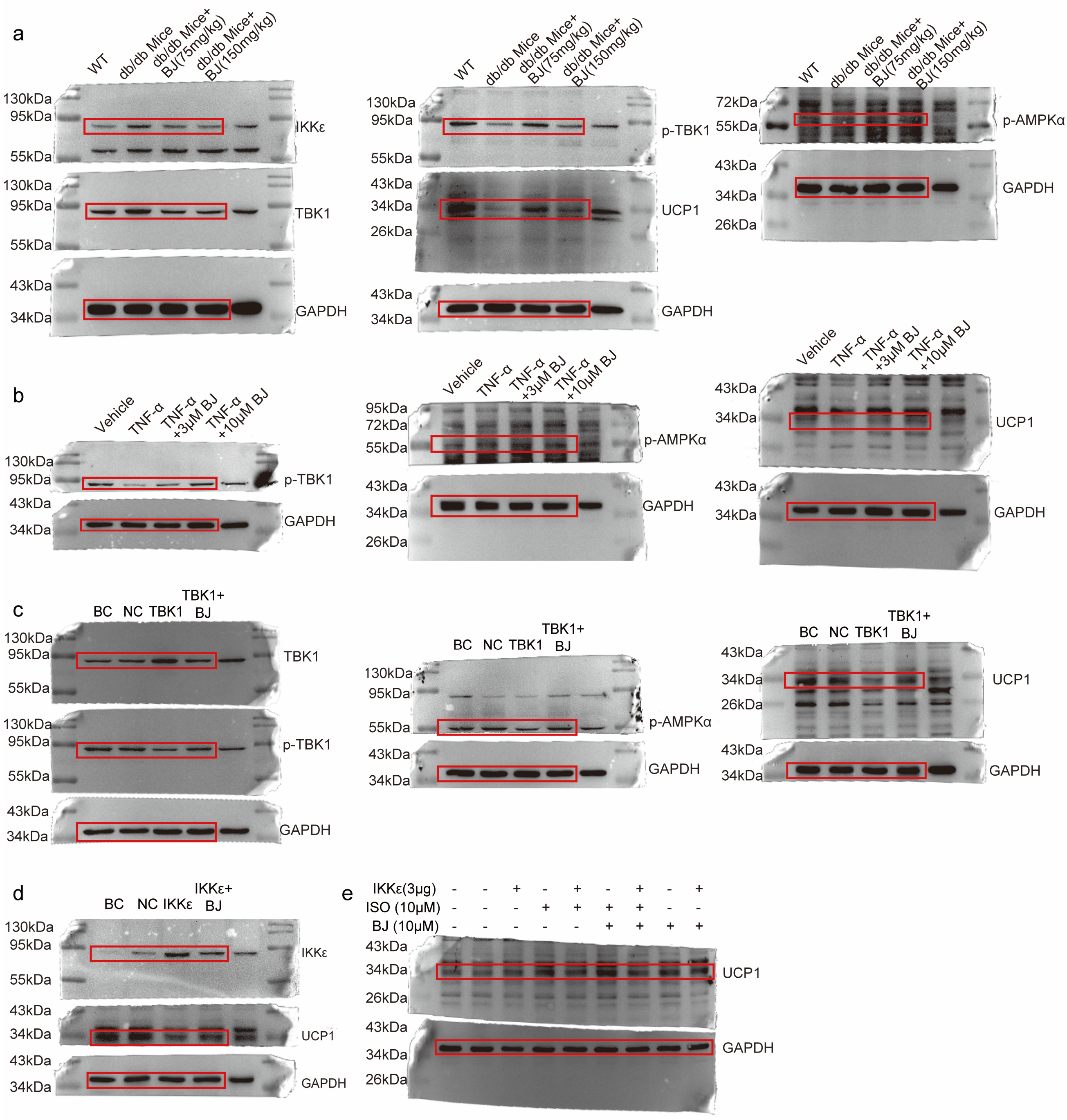

Supplementary Figure 12. (a) Uncropped western blots of Figure 6f. (b) Uncropped western blots of Figure 7i-k. (c) Uncropped western blots of Figure 8a, 8c and 8d. (d) Uncropped western blots of Figure 8h. (e) Uncropped western blots of Figure 8j.

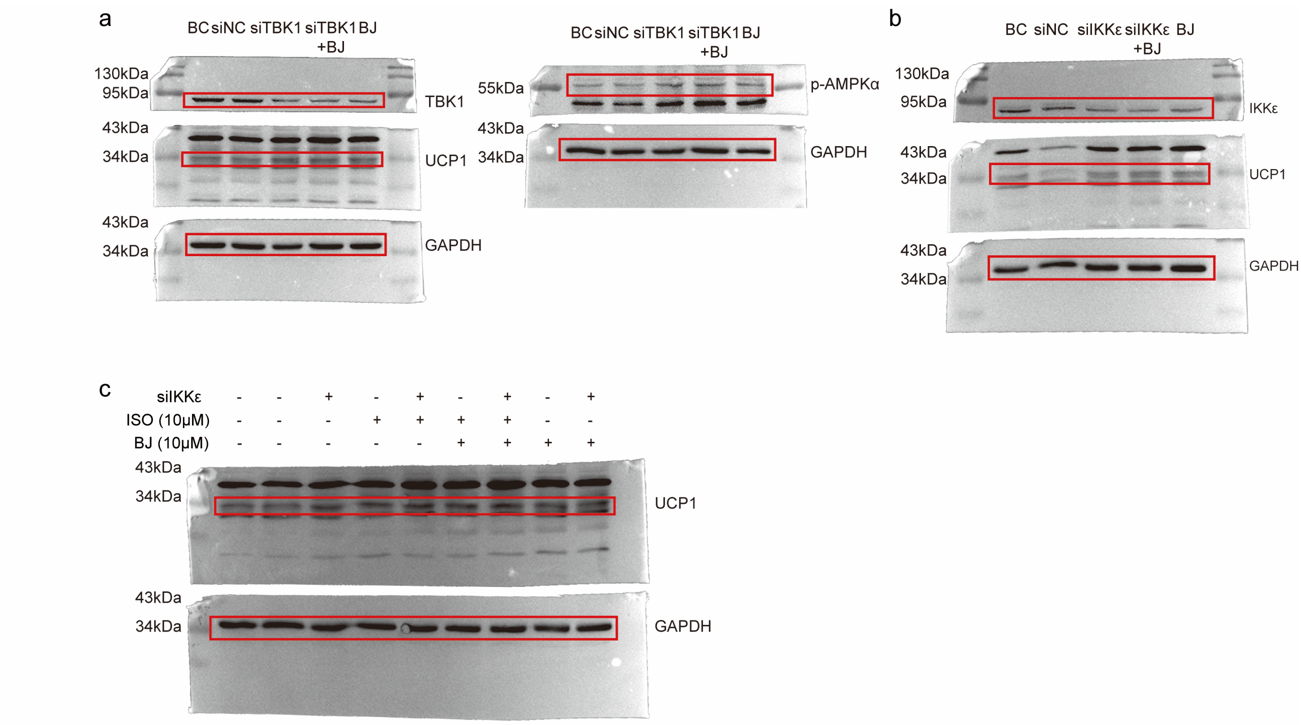

Supplementary Figure 13. (a) Uncropped western blots of Figure 9a. (b) Uncropped western blots of Figure 9h. (c) Uncropped western blots of Figure 9j.
