# Supplementary material for: A co-crystal berberine-ibuprofen improves obesity by inhibiting the protein kinases TBK1 and IKKɛ
Source: Commun Biol. 2022 Aug 12;5:807. doi: 10.1038/s42003-022-03776-0 (PMC9374667; doi:10.1038/s42003-022-03776-0)
Supplement: Supplementary file 3 — Description of Additional Supplementary Files [file 42003_2022_3776_MOESM3_ESM.pdf]

Description of Additional Supplementary Files

File name: Supplementary Data 1
Description: The source data behind the graphs in the main article and supplementary information.
